# Heat Storage of Paraffin-Based Composite Phase Change Materials and Their Temperature Regulation of Underground Power Cable Systems

**DOI:** 10.3390/ma14040740

**Published:** 2021-02-05

**Authors:** Peiling Xie, Haoliang Huang, Yuchang He, Yueyue Zhang, Jiangxiong Wei

**Affiliations:** School of Materials Science and Engineering, South China University of Technology, Guangzhou 510640, China; xplgreat@163.com (P.X.); huanghaoliang@scut.edu.cn (H.H.); hycperfect@163.com (Y.H.); msyyzhang@163.com (Y.Z.)

**Keywords:** porous ceramsite, expanded graphite, paraffin-based CPCMs, underground power cable system, temperature regulation

## Abstract

Excessive heat accumulation in backfill materials causes thermal fatigue damage in underground power cable systems that significantly affects the cable carrying capacity. To improve the thermal conditions of the system, two types of composite phase change materials (CPCMs) were prepared by incorporating paraffin into porous ceramsite (CS)/expanded graphite (EG) in this study. EG and CS can carry 90 and 40 wt.% paraffin, respectively. The phase change temperature of paraffin/CS and paraffin/EG CPCMs was approximately 65 °C, and the corresponding latent heats were 63.38 J/g and 156.4 J/g, respectively. Furthermore, the temperature regulation by CPCMs was evaluated experimentally by designing a setup to simulate the underground power cable system. The reduction in the maximum temperature of the backfill materials with paraffin/CS CPCM and paraffin/EG CPCM was approximately 7.1 °C and 17.1 °C, respectively, compared to reference samples. A similar conclusion was drawn from the heat flux curves. Therefore, the prepared CPCMs could significantly alleviate temperature fluctuations, where the paraffin/EG CPCM provided better temperature regulation than paraffin/CS CPCM. Both materials have potential applications for use in backfill materials for underground power cable systems.

## 1. Introduction

Population growth and an increasing electric energy demand has resulted in the extensive growth of power cable applications. Underground power cable systems (UPCSs) are considerably superior to cable tunnels or overhead transmission lines because of their facile construction, the absence of disturbances to surroundings, and cost savings, and play a dominant role in modern power cable network systems. The safe and efficient operation of underground cables is key to ensuring transportation by power cable systems [1,2].

Cables at their maximum carrying capacity are generally considered to have the highest commercial value. A consideration of Joule’s law shows that the carrying capacity depends on the conductor temperature. For safety considerations, the recommended optimum temperature is 60 °C, and the limiting temperature is 90 °C [3]. However, the dry zones around a cable that usually form under long-term thermal cycling conditions lead to a sharp rise in the temperature of UPCSs. Therefore, the ability to dissipate heat from the cable to its surroundings is an important performance parameter for evaluating a buried cable system [4].

Backfill material is a medium used to transfer heat from cables to the surrounding soil, and is widely used to improve the external heat dissipation environment of cables. The International Electrotechnical Commission (IEC) provides formulas to calculate UPCS current ratings, which show that using surrounding backfill materials with a high thermal resistance results in an increase in operation temperature and prevents an increase in the electrical ampacity [5,6]. The thermal resistance of backfill materials depends strongly on the material moisture content, but water migration is unavoidable under the continuous heat release from cables. Thus, the heat capacity of the backfill material can be increased to effectively manage the UPCS thermal environment.

Phase change materials (PCMs) are a class of energy storage materials based on latent heat mechanisms that can release or absorb heat via phase changes (usually solid–liquid transitions). PCMs exhibit considerable potential for temperature adjustment and thermal management. They are widely applied in many fields, such as smart textiles, water heat, solar energy, and buildings [7,8,9,10,11,12]. In principle, PCM behavior can be used to reduce extreme temperatures and temperature fluctuations in UPCSs, thereby extending the cable life and decreasing maintenance costs; the function and significance have been verified by many studies [13,14,15,16].

There are common methods for PCM to be applied to engineering projects: (1) PCM can be directly added into the backfill materials, but subsequent volatilization, leakage, and corrosion cannot be neglected. Meanwhile, volume changes in the PCM during phase transitions increase the possibility of deformation of backfill materials, leading to poor compatibility [17,18,19]. (2) Microencapsulated PCM techniques have been proposed over the years, but the high production costs and low thermal conductivity of PCMs must be considered for engineering applications [20]. (3) PCMs can also be combined with microporous materials (carrier matrix materials), such as diatomite, expanded perlite, montmorillonite, and metal foams to produce composite PCMs (CPCMs) to effectively combine structural and functional performance [21]. Many studies [22,23,24,25,26] have shown that paraffin/ceramsite (CS) and paraffin/expanded graphite (EG) CPCMs improve PCM thermal stability or thermal conductivity while advancing practical engineering applications.

Some studies have shown the application of PCMs to the construction of ground heat exchangers, a condition similar to UPCSs, for which backfill materials are needed to transmit energy [27,28,29]. The large latent heat absorption and clear diminishing of temperature variations during phase transformation are essential for alleviating the impact of UPCSs on the soil temperature field. Furthermore, the intermittent operation of UPCSs on a regular daily schedule facilitates the operation and recovery of the PCM phase process for each cycle, which is very important for long-term stable system operation [30,31]. However, existing studies on cable backfill materials have mostly focused on increasing the thermal conductivity, such that there have been no significant improvements in either the heat accumulation or the heat capacity of these materials. The use of suitable CPCMs to control the thermal environment represents a novel direction in the UPCS field, whereby the thermal properties and stability of CPCMs could be improved for cable engineering.

In this study, paraffin was used as an energy storage substance for a UPCS and incorporated into two carriers with abundant porous structures, namely, ceramsite (CS) and expanded graphite (EG). The morphological characteristics, thermal properties, and reliability of the prepared composite PCMs (CPCMs) were investigated in detail. On this basis, CPCMs were added as a functional component into backfill materials to increase the materials’ heat capacity and reduce the temperature fluctuations of the UPCS. In addition, a UPCS-like physical model was constructed to evaluate the temperature regulation by CPCMs.

## 2. Materials and Methods

### 2.1. Raw Materials

Paraffin (min 98% pure, industrial grade) was purchased from Shanghai YiYang Instrument Co., Ltd. (Shanghai, China) and its thermophysical properties are shown in Table 1. Paraffin offers the advantages of a high storage heat capacity, good chemical stability, low corrosion, and low cost.

Commercially available porous ceramsite (CS) (Gardening Co., Ltd., Guangzhou, China) was used as a support material to prepare the CPCMs, and the chemical composition and basic physical characteristics of CS are presented in Table 2. The strong adhesion and stable physical properties of CS promote the loading of paraffin, a supporting material used in this study [32].

Expanded graphite (EG) was produced by drying expandable graphite (Graphite Co., Ltd., Qingdao, China) with an expansion rate of 250 mL/g in an oven at 80 °C for 24 h, followed by rapid expansion in a high-temperature furnace.

Epoxy resin (ER) (Chemical Co., Ltd., Chengdu, China) and hardener were then used to encapsulate the CPCM to prevent leakage [33].

Bentonite, sand, ordinary Portland cement, and some additives were purchased from local merchants(Minerals Co., Ltd., Guangzhou China) in Guangzhou for use as backfill materials, and the composition of the cable filling paste in a blender was adjusted to obtain a low initial viscosity and good thermophysical properties, as shown in Table 3 [34,35].

### 2.2. Sample Preparation

#### 2.2.1. Paraffin/CS CPCM

The CS was prepared for use in the paraffin/CS CPCM by washing to remove impurities within the pores, followed by complete drying and sieving. Paraffin was incorporated into the CS by the vacuum impregnation method. A prescribed quantity of CS was placed in a conical flask, to which completely melted liquid paraffin was added in excess to cover the CS under vacuum conditions. When no more bubbles appeared around the CS, the samples were placed in a sieve to remove excess liquid PCM. The samples were cooled, thus completing the CPCM preparation.

**Figure 1:**
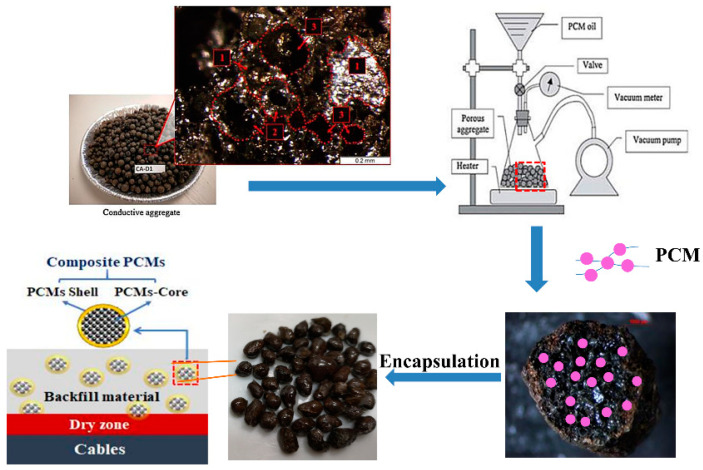
Schematic of the preparation of the encapsulated paraffin/CS composite phase change material (CPCM).

Then, epoxy resin was mixed with a curing agent in a 3:1 ratio and used to coat the paraffin/CS CPCM. The samples were cured for 12 h to yield encapsulated paraffin/CS CPCM. A schematic of the preparation of encapsulated paraffin/CS CPCM is shown in Figure 1.

#### 2.2.2. Paraffin/EG CPCM

**Figure 2:**
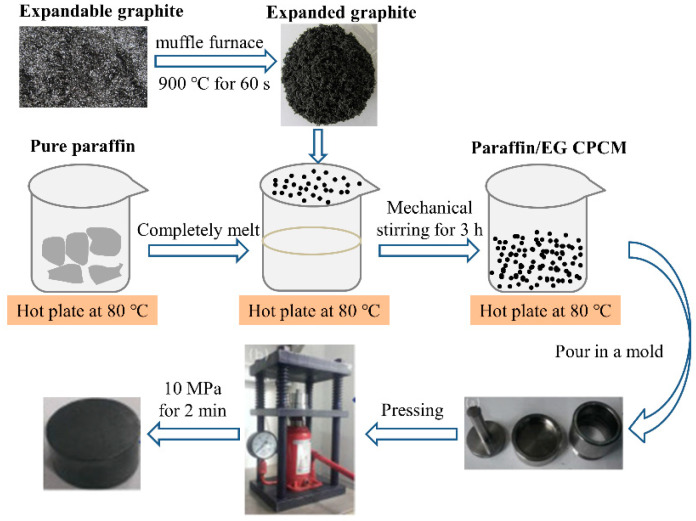
The fabrication process of the formable paraffin/EG CPCM.

Next, melting impregnation was used to prepare paraffin/EG CPCM: EG was added to completely melted liquid paraffin, and the mixture was stirred mechanically for 3 h to ensure that the paraffin entered the EG pores and the composite was uniformly mixed. The paraffin was fully absorbed by the EG for a mixture with 10 wt.% EG, in accordance with the results of Xia et al. [36]. The composite powders were cooled and compressed into a cylinder with a diameter of 10 mm, height of 10~12 mm, and density of 1.83 g/cm^3^ by dry pressing in a mold. A pressure of 5 MPa was applied for 120 s to form samples for thermal stability measurements and use as backfill material. The fabrication process of the formable paraffin/EG CPCM is shown in Figure 2.

#### 2.2.3. Backfill Materials with CPCMs

The backfill materials were prepared in a blender, and were comprised of components such as bentonite, sand, ordinary Portland cement, and some additives, as previously reported [35]. Paraffin/CS CPCM and paraffin/EG CPCM were then added to the backfill materials for cable filling. The obtained composites were poured into the model and maintained under ambient air for 24 h. This filling medium had good fluidity in the initial state and solidified into a gel with good water retention and low thermal resistance after 24 h.

### 2.3. Characterization of CPCMs

The morphological characteristics of CS, EG, and the filling characteristics of CPCMs were observed by scanning electron microscopy (SEM, EVO 18, ZEISS, Jena, Germany). The paraffin load content and the thermal stability of the CPCMs were studied by thermogravimetry (TG, 182 449 C, Netzsch STA, Berlin, Germany) with heating from 25 °C to 600 °C at a heating rate of 10 °C/min in an air atmosphere. The thermal properties were measured by differential scanning calorimetry (DSC, 182 449 C, Netzsch STA, Berlin, Germany). Scanning was conducted at a heating rate of 10 °C/min from 25 °C to 90 °C in an N_2_ atmosphere. The latent heat was obtained by using numerical integration to calculate the area under the peak of the DSC curve.

### 2.4. Thermal Reliability Test of CPCMs

A self-designed leakage test was used to measure the thermal reliability of the CPCM samples. Colored markers were used to draw circles on the center of the filter papers. The same quantity of the samples was placed on filter papers, which were then placed in an oven at 90 °C for 30 min to ensure that the phase change had taken place. The filter papers were removed and cooled to room temperature, and the traces of liquid paraffin on each filter paper were observed. Three parallel samples were taken for each test, and the average result was used to reduce error.

### 2.5. Evaluation of Temperature Regulation Effect

#### 2.5.1. Test Set-Up

**Figure 3:**
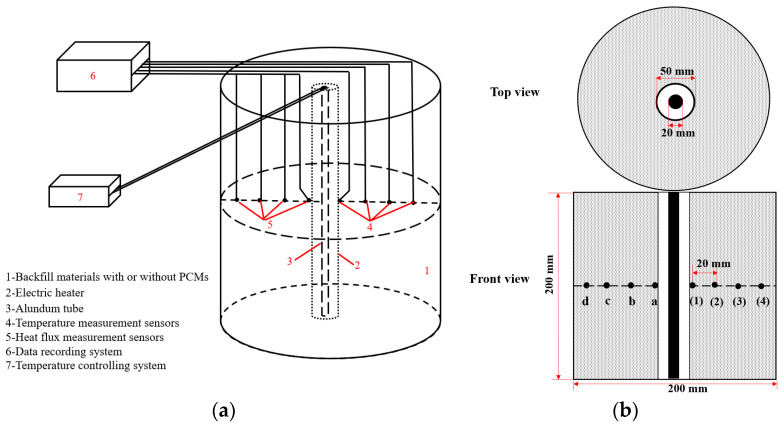
Self-designed setup: (**a**) Schematic; (**b**) Top view of the cylinder with sensors.

Figure 3a shows the self-designed experimental setup that simulates actual UPCS working conditions that were used to study temperature regulation by cable backfill materials containing CPCMs. This apparatus was used to analyze the heat transfer process between the surrounding backfill materials containing CPCMs and the electrical conductor.

The setup was equipped with a heat insulation cylinder, a thermal source, a voltage regulator, and recording systems. These test facilities are described below.

1. The heat insulation cylinder had approximate dimensions of 200 mm × 220 mm (diameter × height) and was made of polyvinyl chloride. The cylinder was initially open at the top and was then filled with backfill materials and sealed with the surrounding soil. The cylinder exterior was wrapped with tinfoil to create an insulation boundary against the outside environment.
2. The thermal source consisted of an electric heating tube (D = 20 mm, 200 W) placed inside an alundum tube (D = 50 mm, Al_2_O_3_ > 98%, excellent heat conductivity) for protection. The source was placed on the symmetry axis of the major side of the container to facilitate even heat release.
3. The voltage regulator was used for temperature control to prevent the cable operating temperature from exceeding 90 °C. Temperature control for the heating conductor was thus realized.
4. The temperature recording system included four temperature sensors, four heat flux sensors, and a temperature recorder. All the sensors were contact-type sensors and were used to measure the temporal evolution of the temperature and heat flux, where the sensor distribution is presented in Figure 3b. This area was thermally monitored by eight sensors evenly spaced over a distance of 20 mm. Temperature thermocouples (K-style) were purchased from Guangzhou QianHui Instruments Co., Ltd., Guangzhou, China. The thermoresistances (JZRL-2) used to measure the heat flux were supplied by TINEL Environment Energy Instruments Co., Ltd., Liaoning, China and operate on the principle of electric heating. The data recorder has 12 channels and can accurately capture the temperature and heat flux signals at the same time. The entire experimental setup was located inside a thermostatic environment and insulated against the external environment.

#### 2.5.2. Experimental Process

The experimental device was assembled as described above, and the CPCMs were used as a functional component to substitute 10 wt.% of the backfill materials for samples B# (added paraffin/CS CPCM) and C# (added paraffin/EG CPCM) to characterize the temperature regulation by the materials relative to that by the original backfill materials without CPCMs (samples A#). Samples A# corresponded to the control models, whereas samples B# and C# corresponded to the experimental models.

The cable backfill materials with/without CPCMs were first placed in a constant-temperature environment. Then, an input voltage (50 V) of the electric heating tube was applied and maintained to steadily produce and conduct the heating power. The temperature and heat flux change of each sensor were tested during the heating/cooling process, which was scheduled as 6 h of heating and 18 h of cooling.

## 3. Results

### 3.1. Morphological Characteristics

**Figure 4:**
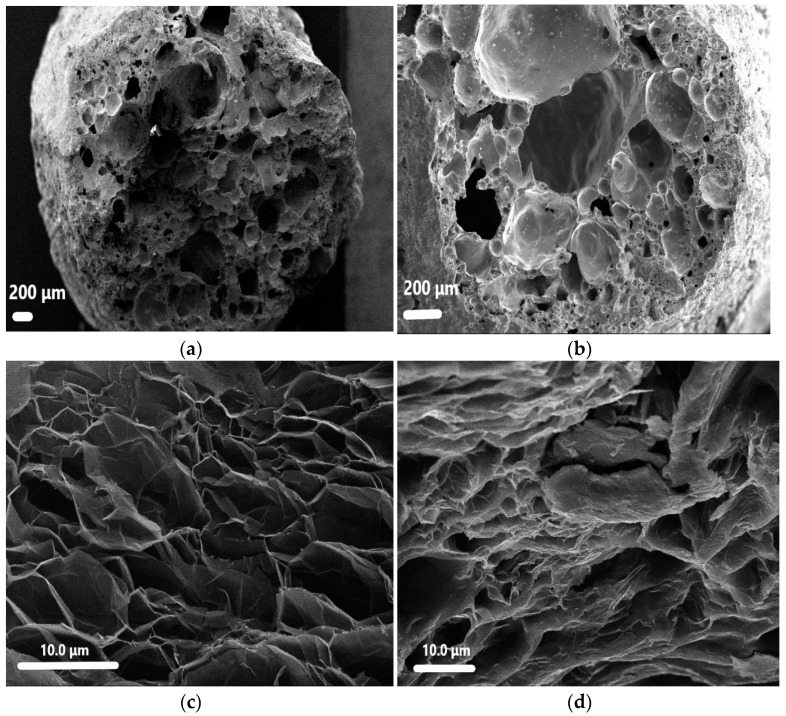
SEM images of samples: (**a**) CS; (**b**) paraffin/CS; (**c**) expanded graphite (EG); (**d**) paraffin/EG.

The morphology and sections of CS and paraffin/CS CPCM were observed by SEM. Figure 4a shows numerous connected and closed pores in the CS. The CS pore structure provides an excellent framework for the PCM that effectively prevents leakage [37]. Figure 4b shows that most of the pores were filled or covered by PCM, indicating that paraffin and CS were successfully combined by the vacuum impregnation method.

The SEM images of EG and paraffin/EG CPCM are displayed in Figure 4c,d. Figure 4c shows numerous interconnected open micro-honeycomb pores in the structure that enable paraffin adsorption. Figure 4d shows an image of the paraffin/EG CPCM, in which the pore structure is mostly covered with paraffin, demonstrating good compatibility between EG CPCM and paraffin. The adsorption properties of paraffin/EG CPCM were influenced by the unique network porosity of EG [38].

### 3.2. Load Capacity of Paraffin on CPCMs and Thermal Stability

**Figure 5:**
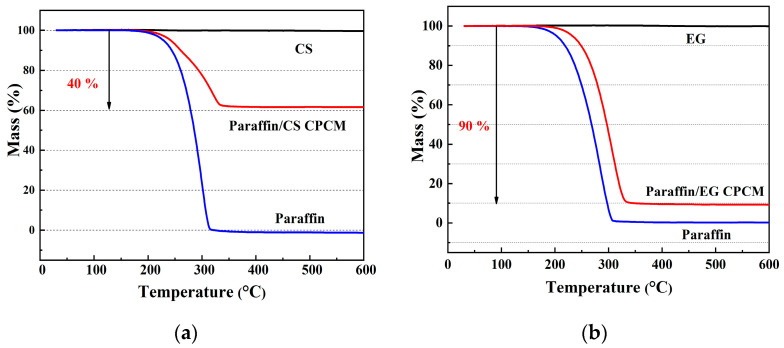
Thermogravimetric (TG) curves of samples: (**a**) paraffin/CS; (**b**) paraffin/EG.

Figure 5 shows the TG curves of CS, paraffin, paraffin/CS, and paraffin/EG CPCMs that were used to quantitatively analyze the load capacity of paraffin on the CPCMs. The curves shows that there was a clear mass loss of paraffin and the CPCMs during the experimental process with little CS and EG loss: these results were attributed to the volatilization of paraffin and the excellent thermal stability of the matrix materials.

The paraffin mass fraction in the CPCMs is denoted by x. The paraffin mass fraction can be calculated by the formula a(1 − x) + bx = c, where a, b, and c are the mass losses of CS/EG, paraffin, and the CPCMs, respectively. The observation that a = 0 and b = 1 in the curves indicates that the total mass losses of the CPCMs were almost equal to the paraffin load capacity on the CPCMs (i.e., c = x). The paraffin load capacity was determined to be approximately 40% for the paraffin/CS CPCM (Figure 5a) and approximately 90% for the paraffin/EG CPCM (Figure 5b). The higher paraffin load ratio for the paraffin/EG CPCM in comparison to the paraffin/CS CPCM resulted from EG being lighter in weight than CS.

The TG curves can also be used to evaluate the thermal stability for the application of CPCMs in UPCSs. Figure 5 shows a similar overall trend in the paraffin curves to that of the CPCMs, except for a higher mass loss rate of paraffin, which suggests that the CS/EG pore structure inhibited paraffin volatilization and promoted the thermal stability of the CPCMs. In addition, there were significant weight losses of paraffin and CPCMs over approximately 200 °C to 350 °C, whereas the mass losses of CPCMs below 200 °C were very small, and the cable operating temperature was usually below 100 °C. Thus, the CPCMs remained stable in the UPCS, which promoted durability and temperature regulation.

### 3.3. Thermal Properties of CPCMs

**Figure 6:**
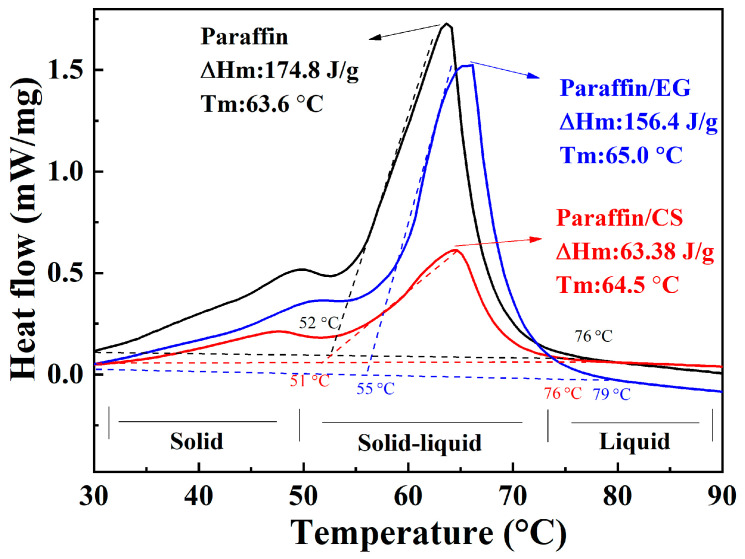
Latent heat of the paraffin and CPCMs.

Figure 6 shows the DSC curves for paraffin, paraffin/CS CPCM, and paraffin/EG CPCM. The curves exhibit three distinct phase regions: the first region corresponds to a solid phase below 50 °C, the second region corresponds to a solid–liquid phase transition at approximately 60 °C, and the third region corresponds to a liquid phase in the melting state above 75 °C. Endothermic sample peaks were observed over 50–75 °C, which was consistent with the maximum allowable UPCS operating temperature range.

The phase change temperature (T_f_) and melting enthalpy (ΔH_f_) were obtained by analyzing the DSC curves. The T_f_ and ΔH_f_ of paraffin were 63.6 °C and 174.8 J/g; paraffin/CS CPCM melted at 64.5 °C with a ΔH_f_ of 63.38 J/g, and paraffin/EG CPCM melted at 65.0 °C with a ΔH_f_ of 156.4 J/g. The T_f_ of the CPCMs was higher than that of the corresponding paraffin, which could be attributed to the surface tension and capillary force between paraffin and the CS/EG inner surface. Strong interactions increase T_f_ [39]. The density change after combination could also affect T_f_. The density decreased, and the phase change temperature increased. In addition, the ΔH_f_ values of paraffin/CS and paraffin/EG CPCM decreased to approximately 36.3% and 89.5%, respectively, compared to that of paraffin, which were slightly lower than those calculated from the TG curves. The variation in ΔH_f_ was mainly influenced by the decrease in the percentage of paraffin, and some paraffin was confined in the narrow zone and could not completely melt and crystallize over the considered temperature range [40].

This result indicates a more desirable ΔH_f_ for paraffin/EG than paraffin/CS, which resulted from the higher paraffin mass fraction in the paraffin/EG CPCM than the paraffin/CS CPCM. In conclusion, the two types of CPCMs exhibited good thermal properties, including a large latent heat and a suitable phase change temperature, which promoted heat absorption in the UPCS.

### 3.4. The Exudation Stability of CPCMs

**Figure 7:**
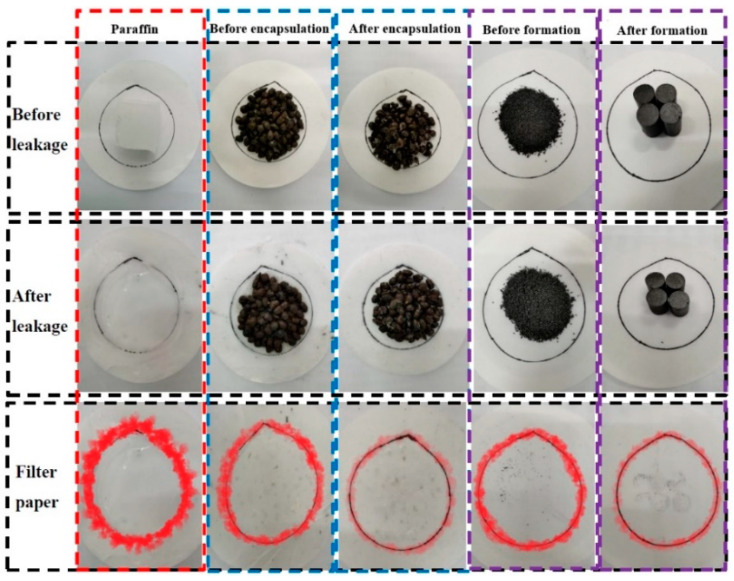
The paraffin leakage region is marked in red for samples before and after heating.

The exudation stability of the samples was characterized by red paraffin traces on the filter papers at the end of the leakage test. Figure 7 shows that most of the molten paraffin reached the edge of filter paper when solid paraffin was the heated sample. When unencapsulated paraffin/CS CPCM was heated, paraffin leakage occurred in the section within the red circle. Little paraffin was observed within the circle for encapsulated paraffin/CS CPCM, suggesting that the encapsulation process can improve the CPCM thermal stability. Similarly, a portion of the paraffin flowed to the edge of the filter paper when paraffin/EG powder was heated. However, a very small quantity of paraffin in the paraffin/EG cylinder was found on the filter paper. The trend in the abovementioned results indicates that the pressed forming process can improve the thermal reliability of paraffin/EG. The overall conclusion is that encapsulation (ER)/formation (dry pressing) can significantly decrease paraffin leakage to improve the long-term thermal reliability [33,38].

### 3.5. Temperature Regulation Effect of the Backfill Materials with CPCMs

**Figure 8:**
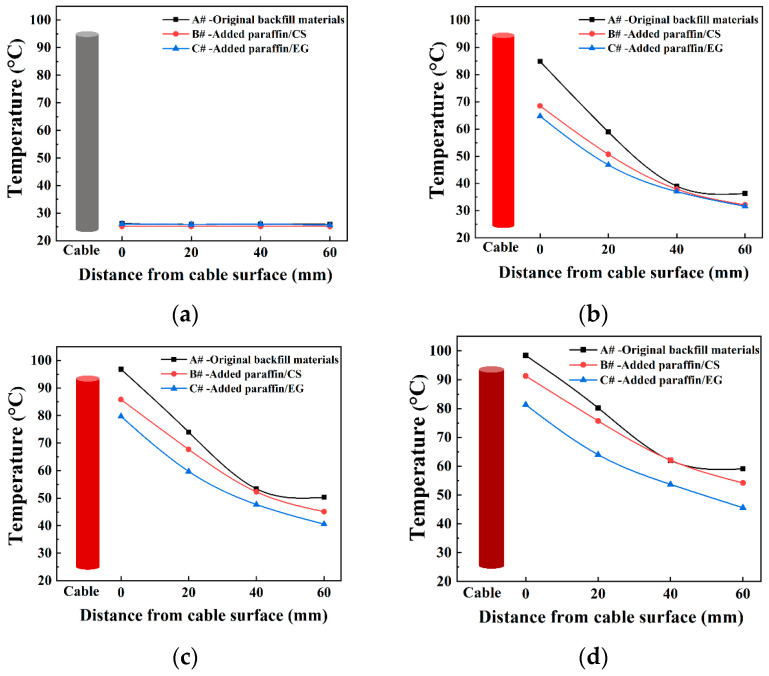
Temperature variations of the samples during different heating periods: (**a**) T = 0 h; (**b**) T = 2 h; (**c**) T = 4 h; (**d**) T = 6 h.

The abovementioned experimental results demonstrate the desirable properties of the paraffin/CS and paraffin/EG CPCMs. Hence, the CPCMs were used as a functional component to replace a prescribed quantity of backfill materials to prepare samples B# (added paraffin/CS CPCM) and C# (added paraffin/EG CPCM), which were used in conjunction with control model samples A# (the original backfill materials) to characterize the temperature regulation ability of the materials. Figure 8 shows the recorded temperature variations of the sensors set at different distances from the heat source for samples A#, B#, and C# during the heating period. Similar temperature variation curves were obtained for the three samples, and the sample temperatures changed with time, which was consistent with the theoretical heat transfer process under constant heat flow [41].

All the temperature readings increased rapidly with time during the initial stage of heat exchange and then rose gradually to reach a stable temperature range. The closer the measuring sensor was to the heat source, the more drastic the temperature fluctuation was. The intensity of the fluctuation decreased with increasing distance from the measurement point to the cable, as shown in Figure 8a–d. The temperatures eventually attained a steady value when the heating time reached 6 h. The equilibrium temperature of sensor (1) for samples A# was significantly higher than that of samples B# and C#. The maximum temperature reduction between samples A# and B# reached approximately 7.1 °C, whereas that between samples A# and C# reached approximately 17.1 °C.

These results indicate that the maximum limiting temperature of the original backfill materials was easily attained, whereas the added CPCMs absorbed heat and reduced the rising temperature rate of the surrounding cable, especially for samples C#. The paraffin/EG CPCM had the highest volume heat storage capacity and heat absorption rate. The good thermal conductivity of EG can improve the temperature sensitivity of CPCMs and accelerate the phase change process [42]. Consequently, the addition of CPCMs can effectively improve heat accumulation around cables and alleviate the effects of the surrounding soil temperature.

**Figure 9:**
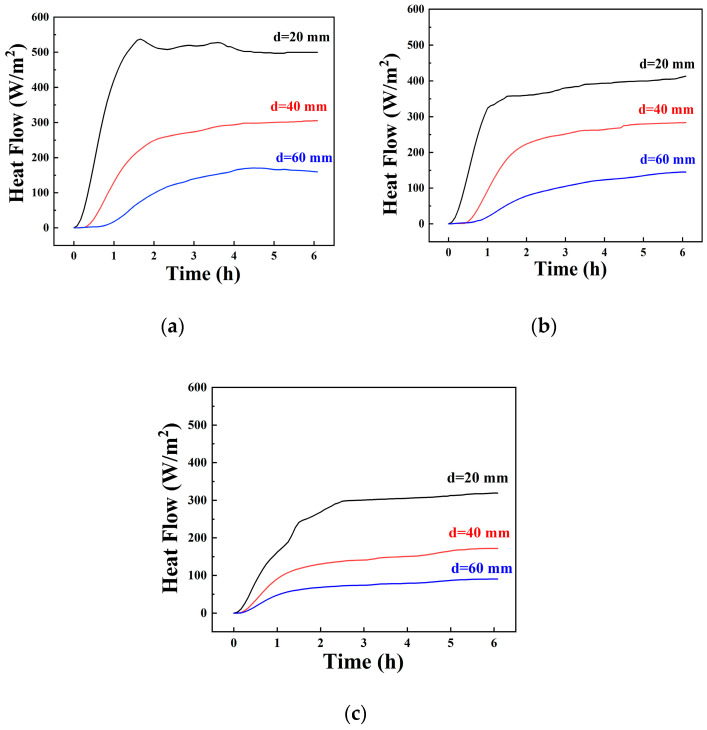
The heat flux variations of samples at different distances d between the heat source and sensors: (**a**) samples A#, (**b**) samples B#, (**c**) samples C#.

The heat flux q is defined as the heat energy passing through a unit area per unit time during heat conduction. Figure 9a–c shows the recorded heat flux variations in the samples during the heating period. Similar trends are observed for the heat flux variation curves, where the heat flux decreased with increasing distance of the measurement point from the heat source. The heat flux eventually reached a steady value when a constant heat flow was reached. The equilibrium heat fluxes of samples A# at each sensor were clearly higher than those of samples B# and C#. These results correspond to the temperature distribution in Figure 8, which further confirmed the function of the CPCMs in the UPCS. Some disturbances in the heat flux were observed at a 20 cm distance between the sensors and the heat source during the initial heating period because of unstable heat transfer at the site closest to the heat source, which, however, had little effect on the equilibrium heat flux results.

Thus, it can be concluded that the two types of CPCMs added to the original backfill materials could reduce the temperature fluctuations of UPCSs in actual cable engineering applications. The added CPCMs both reduced the peak electric load and decreased the electrical peak–valley difference to protect the cables. Although the paraffin/EG CPCM clearly exhibited superior thermal performance and cooling effects and is suitable for high-loading cable environments with severe thermal concentration, the high cost and relatively complex manufacturing process of this material must be considered in practical engineering applications. Comprehensive consideration of different heat dissipation scenarios during underground cable laying is required to choose suitable CPCMs to meet engineering needs.

## 4. Conclusions

Two types of composite phase change materials (CPCMs) were prepared and characterized in this study as a functional component for backfill materials to reduce thermal fatigue damage to underground power cable systems. The results of this study confirmed the compatibility of paraffin with porous ceramsite (CS) and expanded graphite (EG). The paraffin load capacity was 40 and 90 wt.% for paraffin/CS CPCM and paraffin/EG CPCM, respectively. The TG analysis showed that the CPCMs remained stable below 200 °C and are suitable for underground cable engineering applications. The phase-change temperature of paraffin/CS and paraffin/EG CPCMs was approximately 65 °C, and the corresponding latent heats were 63.38 J/g and 156.4 J/g, respectively. A leakage test was designed to evaluate the properties of the CPCMs, and the test results showed superior exudation stability performance for the CPCMs after encapsulation and formation. Given the good heat storage performance of the CPCMs, the temperature regulation of the CPCMs on underground power cable systems was evaluated using a self-designed cable setup, and the results indicate reductions in the maximum temperature of the backfill materials containing paraffin/CS CPCM and paraffin/EG CPCM of approximately 7.1 °C and 17.1 °C, respectively, compared to reference samples. The results of a heat flux test led to the same conclusion on the effect of added CPCMs on heat transfer. Thus, CPCMs exhibit considerable potential for cable engineering applications.

## Figures and Tables

**Table 1:** Thermophysical properties of paraffin.

Material	Melting Point (°C)	Density of Liquid Phase (g/cm^3^)	Density of Solid Phase (g/cm^3^)	Specific Heat (kJ/m^3^·K)	Thermal Conductivity (W/m·K)
Paraffin	60–64	0.768	0.900	1635	0.405

**Table 2:** Chemical composition and physical characteristics of porous ceramsite (CS).

**Chemical Compositions (wt.%)**										
**SiO_2_**	**K_2_O**	**Fe_2_O_3_**	**MnO**	**P_2_O_3_**	**SO_3_**	**Al_2_O_3_**	**Na_2_O**	**TiO_2_**	**CaO**	**MgO**
52.92	2.83	13.39	0.45	0.19	0.15	20.84	0.66	0.95	6.13	0.99
**Physical Characteristics of Porous CS**										
**Cylinder Compressive** **Strength (MPa)**			**Water Absorption (%)**			**Bulk Density** **(g/cm^3^)**			**Diameter Range** **(mm)**	
2.20			11.00			0.40			3.0–5.0	

**Table 3:** Basic physical properties of cable backfill material.

Fluidity (mm)	Initial Viscosity (Pa·s)	24 h Viscosity (Pa·s)	Thermal Conductivity (W/m·K)	Specific Heat (kJ/m^3^·K)
185	0.071	0.287	0.77	3626

Note: The fluidity typically reflects the working performance of a paste and is determined using a flow table.

## Data Availability

No new data were created or analyzed in this study. Data sharing is not applicable to this article.
